# Aqueous-Deficient Dry Eye Exacerbates Signs and Symptoms of Allergic Conjunctivitis in Mice

**DOI:** 10.3390/ijms23094918

**Published:** 2022-04-28

**Authors:** Tatsuma Kishimoto, Waka Ishida, Isana Nakajima, Ken Fukuda, Kenji Yamashiro

**Affiliations:** Department of Ophthalmology and Visual Science, Kochi Medical School, Kochi University, Kochi 783-8505, Japan; t.kishimoto@kochi-u.ac.jp (T.K.); wakai@kochi-u.ac.jp (W.I.); jm-i-nakajima@kochi-u.ac.jp (I.N.); yamashk@kochi-u.ac.jp (K.Y.)

**Keywords:** dry eye, allergic conjunctivitis, tear fluid, conjunctiva, cornea, barrier function, mouse

## Abstract

Dry eye disease (DED) and allergic conjunctivitis affect a large number of patients, and many patients usually have both symptoms. We investigated the interactions between DED and allergic conjunctivitis in mice. Four experimental groups were compared: control, DED, allergy, and allergy with DED. DED was induced by removing the extraorbital lacrimal glands of the mice. Allergic conjunctivitis was induced by intraperitoneal administration of ovalbumin and antigen eye drops. The early phase reaction of the allergy was evaluated using the clinical score, scratching behavior, and vascular permeability in the conjunctiva. Epithelial barrier function was assessed by an LC-biotin assay. Tear fluid volume and corneal fluorescein staining decreased in the DED and allergy with DED groups. LC-biotin penetrated the entire epithelium of both the cornea and conjunctiva in DED mice. The clinical score of the early phase reaction was higher in allergy-induced mice than in non-allergy mice. Edema of the eyelid and conjunctiva were aggravated in mice with DED. The number of scratching episodes and leakage of Evans blue into the conjunctiva were higher in allergy-induced DED mice than in control mice. The presence of aqueous-deficient dry eye caused ocular surface epithelial damage and exacerbated allergic signs and symptoms.

## 1. Introduction

Allergic conjunctival disease (ACD) is an allergen-induced inflammatory disease of the conjunctiva. Several types of ACD have been identified: seasonal and perennial allergic conjunctivitis (AC), atopic keratoconjunctivitis, vernal keratoconjunctivitis, and giant papillary conjunctivitis [1]. Allergic diseases are very common worldwide, and their prevalence is increasing. In a recent survey, the prevalence of allergic conjunctival diseases (ACDs) was reported to be 48.7% in Japan [2]. Allergic conjunctivitis is the most common type of ACD and is mediated by IgE-dependent type I hypersensitivity. Atopic keratoconjunctivitis and vernal keratoconjunctivitis, which are more severe forms of ACD, are caused by both IgE-dependent reactions and non-IgE-mediated chronic inflammation. Conjunctival mast cells play critical roles in the conjunctival response in allergic conjunctivitis. Allergens that penetrate the conjunctiva cross-link the antigen-specific IgE on mast cells, causing mast cell degranulation and inducing itching, edema, and hyperemia. Therefore, reduced permeability of the epithelium and clearance of ocular surface antigens may affect signs and symptoms of allergic conjunctivitis.

Dry eye disease (DED) is a multifactorial chronic disease characterized by loss of homeostasis in the tear film [3]. The prevalence of DED is also high worldwide, and its frequency is reported to be higher in Asia, including Japan, than in other continents [4]. Large epidemiological studies have shown that the prevalence of DED in Japan is 17.4% among men and 30.3% among women [5]. In an epidemiological survey of office workers, the prevalence of DED, including probable cases, was very high at 65.6% [6]. Using visual display terminals for >4 h is associated with an increased risk of DED [7], and may be related to the high prevalence of dry eye in Japan. Etiologically, DED can be classified as aqueous-deficient dry eye (ADDE) or evaporative dry eye (EDE). Recent reports have proposed that ADDE and EDE are not mutually exclusive and may exist on a continuum [3]. Older patients, or those with inflammation of the lacrimal gland, androgen deficiency, or presence of systemic drugs, can also be predisposed to lacrimal gland dysfunction. Both ADDE and EDE cause epithelial cell loss and damage that results in the disruption of the epithelial barrier.

Patients with DED and ACD show similar signs and symptoms. For example, the simultaneous occurrence of itchiness and dryness of the eye has been observed in many patients [8], and one report showed that many subjects with moderate-to-severe ocular itch symptoms also had severe symptoms of DED [9]. These studies suggest that DED and ACD not only occur simultaneously but that they also mutually influence onset and severity. The causes of DED in patients with ACD have been studied in detail. Ocular surface inflammation in ACD reportedly affects tear volume, tear film stability, mucin expression, meibomian gland dysfunction, and epithelial phenotype, resulting in DED [10,11,12,13,14,15,16,17,18]. Whereas many studies have demonstrated the impact of ACD on DED, there are few direct studies of the impact of DED on ACD. Researchers have speculated that altered epithelial barrier, decreased surface clearance, and inflammation may be factors that exacerbate ACD in patients with DED [19]. However, no direct studies have demonstrated that DED worsens the ocular allergic symptoms. The aim of this study was to investigate whether ADDE affects or exacerbates the signs and symptoms of ACD in mice.

## 2. Results

### 2.1. Effects of AC on Dry Eye

**Figure 1:**
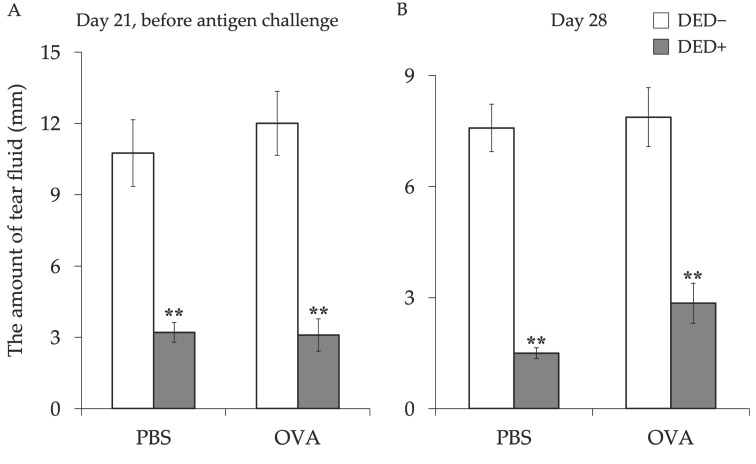
Tear volume before and after induction of AC in mice with or without dry eye (DED). The amount of tear fluid was measured at day 21 before antigen challenge (**A**) and at day 28 (**B**). The length of the wet thread is shown as means ± standard error of the means in each group. Four experimental groups were compared: control (*n* = 6), DED (*n* = 6), allergy (*n* = 6), and allergy with DED (*n* = 5). ** *p* < 0.01 (Tukey–Kramer test) versus non-allergy control group. PBS, mice challenged with phosphate-buffered saline (PBS) (non-allergy group); OVA, mice challenged with ovalbumin (OVA) (allergy-induced group); DED, mice with excised extraorbital lacrimal glands.

**Figure 2:**
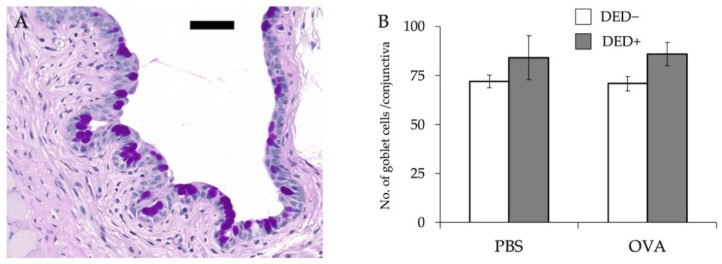
Number of goblet cells in the conjunctiva. The eyes were isolated for histological analysis; the number of goblet cells in the epithelium of the conjunctiva was counted on day 29. Representative photo of conjunctiva and goblet cells showing a purple-magenta periodic acid–Schiff-positive reaction (**A**). Data are shown as means ± standard error of the means in each group (**B**). Four experimental groups were compared: control (*n* = 6), DED (*n* = 6), allergy (*n* = 6), and allergy with DED (*n* = 5). PBS, mice challenged with phosphate-buffered saline (PBS) (non-allergy group); OVA, mice challenged with ovalbumin (OVA) (allergy-induced group); dry eye disease (DED), mice with excised extraorbital lacrimal glands. Bar, 50 μm.

**Figure 3:**
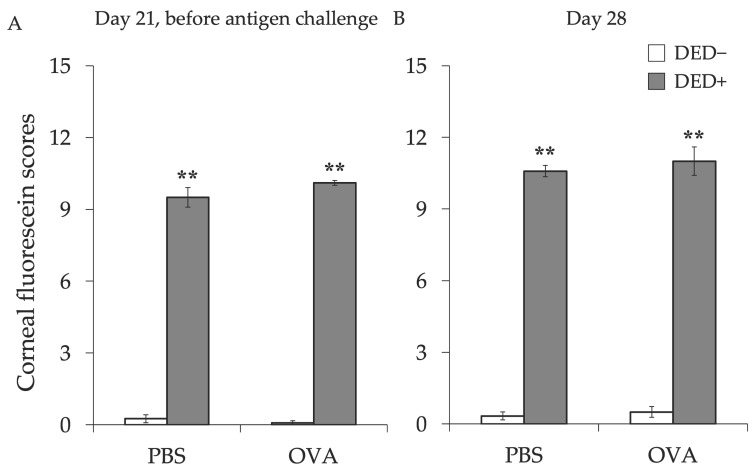
Corneal staining score before and after the induction of AC with or without dry eye (DED). Corneal fluorescein score was classified at day 21 before antigen challenge (**A**) and at day 28 (**B**). The sum of the scores is shown as means ± standard error of the means in each group. Four experimental groups were compared: control (*n* = 6), DED (*n* = 6), allergy (*n* = 6), and allergy with DED (*n* = 5). ** *p* < 0.01 (Tukey–Kramer test) versus non-allergy control group. PBS, mice challenged with phosphate-buffered saline (PBS) (non-allergy group); OVA, mice challenged with ovalbumin (OVA) (allergy-induced group); DED, mice with excised extraorbital lacrimal glands.

First, we examined the effects of AC on dry eye signs. Before lacrimal gland removal, there was no difference in tear fluid volume between the lacrimal gland removal group and control group (data not shown), but the amount of tear fluid was significantly decreased in mice with excised lacrimal glands regardless of the induction of AC (Figure 1). The number of goblet cells in the conjunctiva tended to increase in DED mice, but there were no significant differences between any of the groups (Figure 2). Corneal fluorescein scores were significantly high in mice with excised lacrimal glands, but no further increase was observed in allergy-induced DED mice (Figure 3). Corneal fluorescein scores were similar before and after the allergy challenge. 

**Figure 4:**
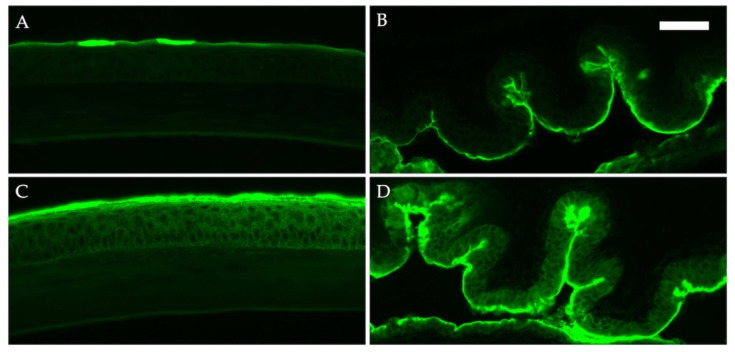
Epithelial barrier function of ocular surface by LC-biotin assay. Epithelial barrier was evaluated by LC-biotin assay. The eyes were challenged with LC-biotin in phosphate-buffered saline 30 min prior to harvest. Frozen sections of the cornea (**A**,**C**) and conjunctiva (**B**,**D**) were stained with streptavidin Alexa Fluor 488 conjugate in mice without (**A**,**B**) and with dry eye (**C**,**D**). Bar, 50 μm.

We also evaluated the barrier function of the ocular surface in DED mice using an LC-biotin assay. Fluorescent immunostaining after the administration of LC-biotin eye drops showed that LC-biotin remained on the superficial epithelial layer of the cornea, and the dye did not penetrate the epithelium of mice without DED. In contrast, the dye infiltrated the entire corneal epithelium layer of DED mice. Similarly, whereas LC-biotin was observed only in the superficial layer of the conjunctival epithelium of mice without DED, dye infiltration was observed in the entire conjunctival epithelium of DED mice (Figure 4).

### 2.2. Effects of Dry Eye on AC

**Figure 5:**
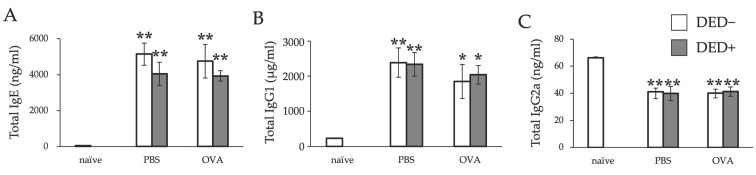
Serum IgE (**A**), IgG1 (**B**), and IgG2a (**C**) levels of mice. Serum IgE and IgG levels were evaluated 24 h after the last antigen challenge. Data are shown as means ± standard errors of the means in each group. Four experimental groups were compared: control (*n* = 6), DED (*n* = 6), allergy (*n* = 6), and allergy with DED (*n* = 5). * *p* < 0.05; ** *p* < 0.01 (Dunnett’s test) versus naïve mice. PBS, mice challenged with phosphate-buffered saline (PBS) (non-allergy group); OVA, mice challenged with ovalbumin (OVA) (allergy-induced group); dry eye disease (DED), mice with extraorbital lacrimal gland excision.

We then examined the effects of DED on AC. Systemic immune responses were not different in mice with or without DED; serum levels of total IgE and IgG1 were significantly increased, and IgG2a was significantly decreased in all sensitized mice, regardless of the presence of DED (Figure 5).

**Figure 6:**
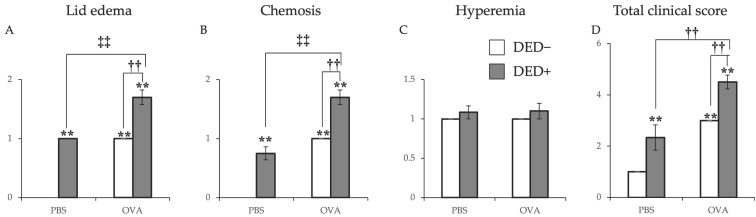
Clinical signs were measured at 20 min after first antigen challenge at day 21. Scores are shown as means ± standard errors in each group. Four experimental groups were compared: control (*n* = 6), DED (*n* = 6), allergy (*n* = 6), and allergy with DED (*n* = 5). ** *p* < 0.01 (Tukey–Kramer test) versus non-allergy control group; †† *p* < 0.01 (Tukey–Kramer test) versus allergy-induced group; ‡‡ *p* < 0.01 (Tukey–Kramer test) versus non-allergy dry eye disease (DED) group.

**Figure 7:**
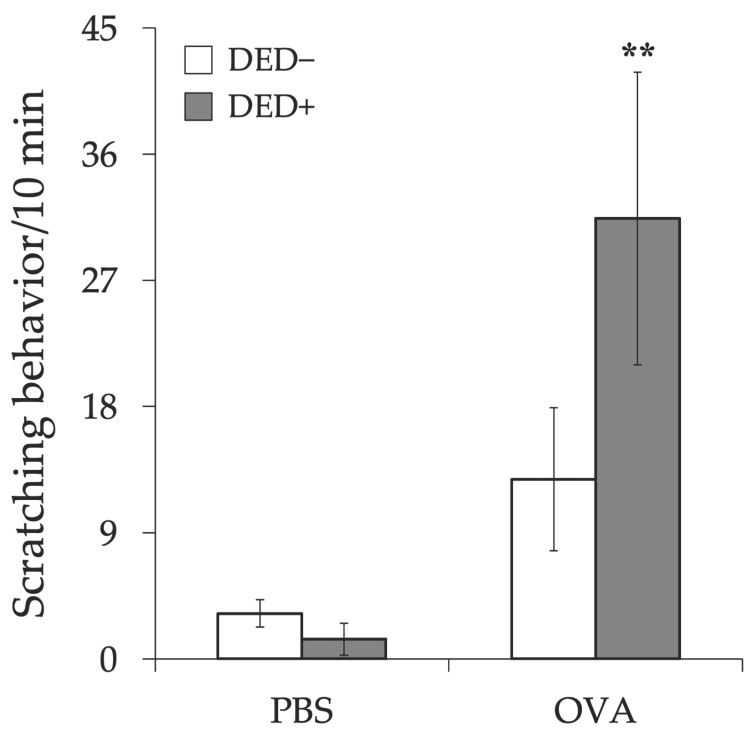
Number of scratching episodes. The scratching behavior of the mice was counted for 10 min after first antigen challenge at day 21, and the number of scratching episodes by the hindlimb was counted. The number of scratching episodes is shown as means ± standard error in each group. Four experimental groups were compared: control (*n* = 6), DED (*n* = 6), allergy (*n* = 6), and allergy with DED (*n* = 5). ** *p* < 0.01 (Tukey–Kramer test) versus non-allergy control group. PBS, mice challenged with phosphate-buffered saline (PBS) (non-allergy group); OVA, mice challenged with ovalbumin (OVA) (allergy-induced group); dry eye disease (DED), mice with excised extraorbital lacrimal glands.

**Figure 8:**
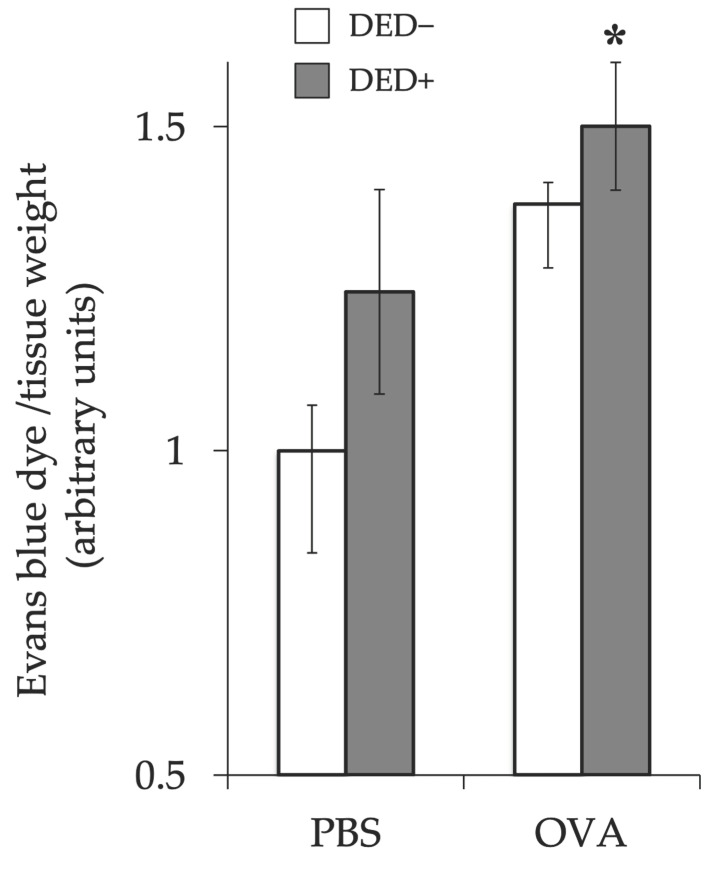
Evans blue dye leakage in conjunctiva. Dye leakage was evaluated 30 min after antigen challenge at day 28, shown as arbitrary units ± standard error in each group. Four experimental groups were compared: control (*n* = 6), DED (*n* = 6), allergy (*n* = 6), and allergy with DED (*n* = 5). * *p* < 0.05 (Tukey–Kramer test) versus non-allergy control group. PBS, mice challenged with phosphate-buffered saline (PBS) (non-allergy group); OVA, mice challenged with ovalbumin (OVA) (allergy-induced group); dry eye disease (DED), mice with excised extraorbital lacrimal glands.

The clinical scores of early phase reactions, including lid edema, conjunctival edema (chemosis), and conjunctival hyperemia, were measured 20 min after the last antigen challenge. Lid edema, chemosis, and total scores were higher in allergy-induced mice than in non-allergic control mice. The clinical score further increased in the allergy-induced DED mice than that in the allergy-induced mice (Figure 6). Scratching behavior after antigen challenge was also increased in allergy-induced DED mice compared to that in non-allergic control mice (Figure 7). The changes in vascular permeability, as early phase reactions of the conjunctiva, were evaluated by the leakage of Evans blue in the conjunctiva. The amount of leakage was significantly higher in allergy-induced DED mice than in non-allergic control mice (Figure 8).

## 3. Discussion

In this study, we demonstrated that tear deficiency exaggerates the signs and symptoms of AC in mice. The signs and symptoms of AC, including edema of the eyelid and conjunctiva, eye scratching behavior, and vascular permeability in the conjunctiva after antigen challenge, were significantly augmented in mice that underwent lacrimal gland removal. Decreased tear clearance and epithelial barrier function of the ocular surface may result in longer retention and higher penetration of antigens to the ocular surface, leading to exaggeration of the symptoms of AC. Epithelial barrier dysfunction can lead to pathogenesis of both DED and ACD [19,20]. Many reports have demonstrated that ACD affects the status of tear fluid and ocular surface epithelia, resulting in DED [10,11,12,13,14,15,16,17,18]. However, there are no basic or clinical studies that directly show the effect of DED on the clinical signs and symptoms of ACD. The present study demonstrates, for the first time in an animal study, that lacrimal ADDE may exaggerate signs and symptoms of ACD. Our study may be a first step toward the development of therapeutic agents and the elucidation of the pathophysiology of patients with combined DED and ACD.

Various ocular surface diseases manifest as impairment of the epithelial barrier, including DED, ACDs, infection, and chemical injuries [21]. Yokoi et al. demonstrated that the corneal epithelial barrier function had significantly decreased in accordance with the severity of superficial punctate keratopathy in DED patients, as measured by fluorescein uptake [22]. In addition, treatment with hyaluronan eye drops alleviated the decline in the corneal barrier in patients with DED [23]. Furthermore, corneal barrier function reportedly decreased even in patients with DED who did not exhibit visible corneal epithelial damage on slit-lamp examination [24]. These studies suggest that epithelial tight junctions of the ocular surface may be impaired in patients with DED regardless of the presence of visible corneal lesions on slit-lamp examination. In mice, dry eye experimentally induced by low humidity environmental challenge also decreased the levels of tight junction proteins and impaired corneal epithelial barrier function [25,26]. In patients with seasonal AC, the expression of the adherens junction protein E-cadherin in the conjunctival epithelium is reportedly downregulated, regardless of the season [1]. Conjunctival allergen challenge also reportedly decreased the expression of the tight junction proteins zonula occludens-1 and E-cadherin in the conjunctival epithelium in a mouse model of AC [11]. Decreased epithelial barrier function is an important factor for the sensitization and exacerbation of allergic diseases. In this study, we demonstrated that mice with tear deficiency had impaired epithelial barriers in the conjunctiva and cornea, as estimated by LC-biotin penetration. Therefore, it is possible that the reduced epithelial barrier function of the conjunctiva allows antigens to penetrate the conjunctiva easily, possibly exacerbating allergic symptoms.

In the present study, because the antigen was systemically sensitized with an adjuvant, a strong Th2-type immune response could be induced; thus, the systemic immune response did not differ between mice with and without dry eye. Fujishima et al. reported that tear volume and clearance were lower in patients with AC who tested negative for serum antigen-specific IgE than in those who tested positive for serum antigen-specific IgE [27]. In a recent study, AC was observed in patients who tested negative for serum antigen-specific IgE [28]. Therefore, in patients with aqueous-deficient dry eye, the antigens may remain on the ocular surface for a long period owing to poor tear clearance, possibly contributing to local sensitization and exacerbation of symptoms.

The influence of different types of ACDs on tear function has been extensively investigated. Most studies have reported that in patients with AC, tear volume does not change, but tear stability estimated by tear breakup time (TBUT) is reduced [10,15,18,27]. In patients with atopic keratoconjunctivitis (AKC), the tear volume has been reported to be unchanged [11,12,16,17], while some studies have reported a decrease in the tear volume [13,14]. However, all those studies reported a decrease in the TBUT [11,12,13,14,16,17]. Onguchi et al. revealed that the onset time of AKC affects tear function and ocular surface findings, and tear volume and epithelial damage in childhood-onset adult AKC patients were considerably worse than those in adult-onset adult AKC patients, pediatric patients, and controls. These results suggest that prolonged ocular surface inflammation may be important for the disruption of the healthy ocular surface, including the tear film [16]. In patients with vernal keratoconjunctivitis (VKC), the tear volume is increased, but the TBUT is reportedly decreased [14,29]. Interestingly, decreased tear film stability has been observed even in the quiescent phases of VKC [29]. Conjunctival goblet cells have also been reported to decrease in patients with various ACDs, including AC [18,30], AKC [13,14,16,17,31], and VKC [14,17]. In particular, a decrease in MUC5 AC, a secreted mucin, has been found along with a decrease in goblet cells in patients with AKC [13,14,16]. In contrast, the density of goblet cells and the severity of corneal staining were not affected in mice with AC in the present study, which may change in the future depending on the duration of ocular surface inflammation, as reported by Onguchi [16].

When cells become necrotic, intracellular inflammatory substances called alarmins, such as IL-1α and IL-33, are released outside the cells, resulting in sterile inflammation [32,33]. Recent studies have reported the involvement of alarmins in both ocular allergies and DED. Alarmins released from necrotic corneal epithelial cells act on corneal fibroblasts and epithelial cells, causing enhanced production of chemokines and cytokines, such as eotaxin, and decreased epithelial barrier function [34,35]. IL-33, an epithelial cell-derived alarmin, does not degranulate mast cells by itself but synergistically degranulates mast cells when acting simultaneously with antigens [36]. Alarmin may be involved in the exacerbation of allergic inflammation [37]. The concentration of IL-33 is also reportedly elevated in the tear fluid of DED patients [38,39]. In addition, the tear levels of IL-33 in patients with DED were positively correlated with the tear levels of IL-4 and IL-5 [38]. These results may be related to the fact that many patients have overlapping symptoms of itching and dryness of the eye [8] and that dry eye is severe in patients with itching [9]. Therefore, alarmins released by epithelial injury caused by dry eye may exacerbate allergic symptoms by promoting mast cell degranulation. The role of alarmins in DED and allergies requires further investigation.

We evaluated the epithelial barrier function with the use of the LC-biotin assay. The limitation of the present study is the lack of detailed analysis of each component of the epithelial barrier, such as the adhesion proteins and the mucin expression. Further immunohistochemical analysis should be performed in the future to evaluate the ocular surface epithelia and the mucin expression in more detail.

In conclusion, the presence of ADDE exacerbated the clinical signs and symptoms of ACD, possibly through ocular surface epithelial barrier disruption and reduced allergen clearance.

## 4. Materials and Methods

### 4.1. Ethical Treatment of Animals

This study was approved by the Kochi University Animal Care and Use Committee (permit number J-70). BALB/c mice were purchased from Japan SLC Inc. (Hamamatsu, Shizuoka, Japan) and maintained under specific pathogen-free conditions at the animal facility of Kochi Medical School. Eight-week-old specific-pathogen-free female mice were used in the experiments. All the procedures were performed in accordance with the Association for Research in Vision and Ophthalmology Statement for the Use of Animals in Ophthalmic and Vision Research.

### 4.2. Experimental Procedure for Lacrimal Grand Excision and Experimental Allergic Conjunctivitis

The mice were anesthetized with an intraperitoneal injection of an anesthetic combination of 0.45 µg/g medetomidine (Domitor; Nippon Zenyaku Kogyo, Tokyo, Japan), 6 µg/g midazolam (Sandoz, Yamagata, Japan), and 7.5 µg/g butorphanol (Vetorphale; Meiji Seika Pharma, Tokyo, Japan). The exorbital lacrimal glands of both eyes were exposed through a careful incision near the anterior ear and excised [40]. Subsequently, talibid ophthalmic ointment 0.3% (Santen Pharmaceutical Co., Osaka, Japan) was applied.

**Figure 9:**
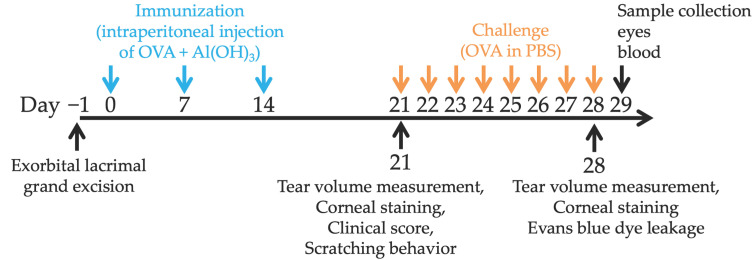
Timeline for induction of dry eye and experimental AC. To induce dry eye, the extraorbital lacrimal glands of the mice were removed 1 day before sensitization. To induce experimental AC, a mixture of ovalbumin (OVA) and alum (Al(OH)_3_) was administered intraperitoneally three times at an interval of 7 days. Seven days after the third sensitization, corneal fluorescein score and the amount of tear fluid were measured; then, both eyes of each mouse were challenged with OVA in phosphate-buffered solution (PBS) from day 21 to day 28.

Experimental AC was induced using a previously described protocol with slight modifications [41] (Figure 9). Briefly, mice were injected intraperitoneally three times with 30 µg ovalbumin (OVA) (Worthington Biochemical Corp, NJ, USA) mixed with 1 mg of Imject Alum (Thermo Fisher Scientific, MA, USA) at intervals of 7 days. Seven days after the third sensitization, both eyes of each mouse were challenged with OVA in PBS (1 mg per 5 µL per eye) from day 21 to day 28.

### 4.3. Evaluation of Dry Eye

To assess dry eye signs, we evaluated corneal fluorescein staining and tear fluid volume as described previously [40]. Fluorescein solution was extracted from one sheet of Fluores Ocular Examination Test Paper 0.7 mg (AYUMI Pharmaceutical Corporation, Tokyo, Japan) with 500 μL of sterile saline. The concentration of the fluorescein sodium salt was approximately 1.4 mg/mL. Then, 1 μL of fluorescein solution was applied to each eye, wiped off, and observed using a portable slit lamp with a blue filter (Kowa Company, Ltd., Tokyo, Japan). Corneal fluorescein scores were classified as 0–3 (0, no fluorescence; 1, sparse spot fluorescence; 2, dense spot fluorescence; and 3, very dense spot fluorescence). Five locations were observed (center, upper left, lower left, upper right, and lower right), and the sum of the scores obtained was calculated. 

The volume of tear fluid was measured using phenol red threads (Zone Quick, Ayumi Pharmaceutical Co. Tokyo, Japan). The mice were fixed for 15 s with the thread inserted into the lower eyelid under no anesthesia, and the length of the wet thread was measured.

### 4.4. Evaluation of Clinical Conjunctival Allergic Reaction

Clinical signs were assessed on day 21, as previously described [42]. The scratching behavior of the mice was counted for 10 min after the first eye drop, and the number of scratching episodes by the hindlimb was counted.

To investigate vascular permeability, Evans blue dye leakage was evaluated as previously described, with slight modifications [43]. The conjunctivas were harvested 30 min after the OVA or PBS challenge. Evans blue was extracted for 48 h in 400 μL of 0.5% Na_2_ SO_4_ and acetone (3:7). After centrifugation, absorbance (620 nm) of the supernatant was measured using a spectrophotometer.

Twenty-four hours after the last challenge, serum IgE, IgG1, and IgG2a levels were evaluated using ELISA, as described previously [41]. OVA-specific IgE and IgG1 were assayed using an anti-OVA IgE ELISA kit or an anti-OVA IgG1 ELISA kit (Cayman Chemical Company, MI, USA) according to the manufacturer’s protocol.

### 4.5. Histological Analysis of Periodic Acid-Schiff Staining, and LC-Biotin Assay

Paraffin sections were cut to 2 µm slices, deparaffinized, and hydrated. The sections were dipped in 0.5% periodate solution (FUJIFILM WakoPure Chemical Corporation, Osaka, Japan), oxidized for 5 min, and then washed with distilled water, dipped in Cold Schiff’s Reagent (FUJIFILM WakoPure Chemical Corporation) for 15 min, washed in water for 5 min, and then placed in sulfurous acid water (FUJIFILM WakoPure Chemical Corporation) for 3 min three times. After rinsing, the sections were dipped in Mayer’s hematoxylin (Muto Pure Chemicals Co., Ltd., Tokyo, Japan) for 5 min. The sections were washed in water for 5 min, dehydrated, and mounted using a mounting medium. The number of goblet cells in the epithelium of the conjunctiva showing a purple-magenta PAS-positive reaction was counted by two observers in a blinded manner.

To assess the epithelial barrier function, we performed an LC-biotin assay. LC-biotin has been reported to cross-link proteins and not penetrate intact tight junctions, and we followed a previously reported method [44]. LC-biotin (Thermo Fisher Scientific, Waltham, MA, USA) in PBS (1 mg/mL; 5 µL) was administered 30 min prior to eye harvest. Frozen sections were cut into 7 µm slices and fixed with 4% paraformaldehyde (Nacalai Tesque, Inc., Kyoto, Japan) for 10 min at room temperature. The cells were then washed with PBS and incubated with streptavidin Alexa Fluor 488 conjugate (Thermo Fisher Scientific) for 1 h at room temperature, washed with PBS, and mounted with the VECTIRSHIELD mounting medium containing DAPI (Vector Laboratories, Inc., Burlingame, CA, USA).

### 4.6. Statistical Analysis

Data were analyzed using Dunnett’s test or the Tukey–Kramer test using Statcel 4 software (OMS, Saitama, Japan).

## Data Availability

Not applicable.
